# Utilizing Instagram Data to Identify Usage Patterns Associated With Schizophrenia Spectrum Disorders

**DOI:** 10.3389/fpsyt.2021.691327

**Published:** 2021-08-16

**Authors:** Katrin Hänsel, Inna Wanyin Lin, Michael Sobolev, Whitney Muscat, Sabrina Yum-Chan, Munmun De Choudhury, John M. Kane, Michael L. Birnbaum

**Affiliations:** ^1^The Zucker Hillside Hospital, Northwell Health, Glen Oaks, NY, United States; ^2^Feinstein Institute for Medical Research, Northwell Health, Manhasset, NY, United States; ^3^Cornell Tech, Cornell University, New York, NY, United States; ^4^Department of Psychology, Hofstra University, Hempstead, NY, United States; ^5^School of Interactive Computing, Georgia Institute of Technology, Atlanta, GA, United States; ^6^Donald and Barbara Zucker School of Medicine at Hofstra/Northwell, Hampstead, NY, United States

**Keywords:** serious mental illness, schizophrenia spectrum disorder, social media markers, digital biomarkers, image analysis

## Abstract

**Background and Objectives:** Prior research has successfully identified linguistic and behavioral patterns associated with schizophrenia spectrum disorders (SSD) from user generated social media activity. Few studies, however, have explored the potential for image analysis to inform psychiatric care for individuals with SSD. Given the popularity of image-based platforms, such as Instagram, investigating user generated image data could further strengthen associations between social media activity and behavioral health.

**Methods:** We collected 11,947 Instagram posts across 68 participants (mean age = 23.6; 59% male) with schizophrenia spectrum disorders (SSD; *n* = 34) and healthy volunteers (HV; *n* = 34). We extracted image features including color composition, aspect ratio, and number of faces depicted. Additionally, we considered social connections and behavioral features. We explored differences in usage patterns between SSD and HV participants.

**Results:** Individuals with SSD posted images with lower saturation (*p* = 0.033) and lower colorfulness (*p* = 0.005) compared to HVs, as well as images showing fewer faces on average (*SSD* = 1.5, *HV* = 2.4, *p* < 0.001). Further, individuals with SSD demonstrated a lower ratio of followers to following compared to HV participants (*p* = 0.025).

**Conclusion:** Differences in uploaded images and user activity on Instagram were identified in individuals with SSD. These differences highlight potential digital biomarkers of SSD from Instagram data.

## 1. Introduction

Schizophrenia Spectrum Disorder (SSD) is one of the leading causes for disability and accounts for an average of 14.5 years of lives lost (1). Despite its low prevalence, the disease burden on patients, families, and society is substantial (2). Early diagnosis and treatment are crucial; however, psychiatric illnesses, including SSD, often receive delayed attention and care resulting in overall worse health outcomes (3, 4). At the same time, the use of digital technologies, such as the internet, smartphones, and social media, is high amongst individuals with serious mental illness (5–7). There is great potential for these technologies to improve outcomes by serving as a source of objective and clinically meaningful collateral patient data, and supporting more informed clinical decision making.

Social media platforms can act as valuable data sources in this process (8, 9). Over the last decade, social media sites, such as Facebook, Twitter, and Instagram, have become increasingly popular. Users engage with these websites to express themselves, share their opinions, and seek social support (6, 10–12). Concurrently, researchers have begun to explore online behaviors on social media and have mapped these behaviors to health-relevant outcomes. This area of focus has ranged from predicting public health issues, e.g., rates of smoking, obesity, and substance-use (13–15), to subjective well-being, including markers of serious mental illnesses and symptomatology (16, 17). In the area of behavioral health, long-term retrospective data from online activity, has demonstrated success in predicting diagnosis (18, 19), prognosis (20–22), and identification of disease-specific usage signals (23, 24).

Instagram is a popular social media platform that has gained traction especially amongst adolescents and young adults (25)—the age groups most at risk for the emergence of SSD. Despite its popularity, the majority of prior research to date has focused on text based platforms such as Facebook (26, 27), Twitter (21, 28), or Reddit (29, 30). Contrary to these other popular networks, Instagram's primary focus is on images and videos as opposed to text (31). It additionally offers easily applicable filters to manipulate photos and videos and to help users express themselves through imagery. Further, Yau and Reich (32) argued that social media provides individuals more time to edit and craft their posts to allow for purposeful and even strategic self-presentation and impression management. In images, meaningful self-expression not just arises from the content, but also composition or camera manipulation, including camera tilt and distance to the object (33, 34). Images can offer particularly interesting insights in psychiatry. For example, it has been argued that researchers and clinicians can learn about their patients behaviors and perception of self through photography and images *via* photo-elicitation (35, 36). Furthermore, Manikonda and De Choudhury (37) found that some individuals use Instagram image posting for mental health disclosures, such as expressing distress, calling for help, and displaying vulnerability. Advancing the field through automated and scalable analysis technologies would facilitate the collection of objective social networking data and support integration into clinical practice.

The majority of prior work, aiming to explore associations between mental health status and social media activity, has been limited by relying primarily on data from individuals with presumed psychiatric conditions, without the ability to validate the disclosure or diagnosis (38, 39). For example, self-disclosures based on specific disease related terms or statements that have been mentioned by platform users (21, 23, 40) or affiliation with mental health related communities (29, 30). Further, social media use of individuals with SSD has been studied little compared to other psychiatric conditions, such as depression, and research has mainly focused on linguistic analysis (39). The use of patients health record data is rare, but necessary, as it yields critical advantages in terms of reliability of the clinical labels.

Our work aimed to investigate Instagram usage patterns of individuals diagnosed with SSD, and to compare them to healthy volunteers (HVs). This is the first study to our knowledge to leverage Instagram data donated directly by psychiatric patients receiving psychiatric care for established and clinically confirmed psychiatric conditions. Using actual patient data is necessary to advance efforts to translate research findings into clinical implementation.

We hypothesized that important differences between participants with SSD and HV exist in their usage patterns on Instagram, including characteristics of posted images, online behavior, and social connections to other platform users.

## 2. Materials and Methods

Participants between the ages of 15 and 35 years, diagnosed with a schizophrenia spectrum disorder, were recruited from The Zucker Hillside Hospital (Northwell Health) inpatient and outpatient psychiatric departments. Most participants with SSD (*n* = 84) were recruited from the Early Treatment Program (ETP), The Zucker Hillside Hospital's specialized early psychosis intervention clinic. Additional participants were recruited from a collaborating psychiatric clinic located in East Lansing, Michigan (HV: *n* = 3; SSD: *n* = 1). Healthy volunteers were also recruited from an existing database of eligible healthy individuals who had already been screened for participation for prior studies and had agreed to be recontacted (*n* = 48). Additional HVs (*n* = 35) were recruited from a large southeastern university in the U.S. *via* an online student community research recruitment site. Healthy status was determined either by the Structured Clinical Interview for DSM Disorders (SCID) conducted within the past 2 years, or the Psychiatric Diagnostic Screening Questionnaire (PDSQ). Written informed consent was obtained for adult participants and legal guardians of participants under 18 years of age. Assent was obtained for participating minors. Participants were fully informed of the potential risks, benefits, and alternative options available, as well as strategies to mitigate risks. Decisional capacity to consent was determined through clinical assessment, as well as *via* completion of a short quiz, designed to assess one's understanding of research procedures, conducted prior to consenting to participate. The study was approved by the Institutional Review Board (IRB) of Northwell Health (the coordinating institution) as well as local IRBs at participating sites.

Participation involved a single study visit. These visits occurred between July 2018 and October 2020. Participants were asked to export their Instagram digital archive by logging on to their Instagram account to request their history. Archives included all retrospective Instagram activity including a history of content posted to the platform (images and associated text), connections to other Instagram users, a list of interactions with other users content (likes and comments) as well as a record of private messages that participants exchanged with other users. Psychiatric diagnoses were ascertained through clinical interviews and were extracted from participants medical records.

### 2.1. Data Processing

The exported Instagram archives were assessed based on inclusion criteria and then processed in a multi-step approach outlined below. An overview of all the extracted features can be found in Table 1.

**Table 1:** Overview of generated features.

**Feature**	**Description**
**General posting usage**	
Duration of use	Time between the first post and the latest post
Posts per month	The total number of posts divided by the duration of use
Posts per time of day	Per participant, the proportion of posts that were done in a certain time window, i.e., night (12 a.m.–6 a.m.), morning (6 a.m.–12 p.m.), afternoon (12 p.m.–6 p.m.), evening (6 p.m.–12 a.m.)
Posts per day of week	Per participant, the proportion of posts that were done on each weekday
**Image features**	
Width	Width of the image in pixels
Height	Height of the image in pixels
Aspect ratio	The ratio of width divided by height. An aspect ratio of 1 characterizes square images.
Percentage of non-square images	Percentage of posts per participant which are not square aspect ratio. Square aspect ratio is the default on the Instagram platform.
RGB/HSV average	For each pixel in an image, we extracted the red, green, blue (RGB) and hue, saturation, value (HSV) color components and averaged these across all pixels. The range for color components apart from hue measures is 0 to 255. Hue has a range from 0 to 179.
RGB/HSV standard deviation	For each pixel in an image, we extracted the red, green, blue (RGB) and saturation, value (HSV) color components and calculated the standard deviation.
RGB/HSV skew	For each pixel in an image, we extracted the red, green, blue (RGB) and saturation, value (HSV) color components and calculated the skew over the histogram. Positive values indicate a skew toward the right of the distribution, i.e., toward higher values, negative values indicate a skew toward the left side of the distribution, i.e., toward lower values.
Colorfulness	A measure of how colorful the image is based on Hasler et al. (49).
Average number of faces	Using the Python OpenCV library and a Haar-Cascade classifier (43), we detected the number of human faces depicted in each image
**Connection statistics**	
Followers	The inwards network connections, i.e., Instagram users who subscribed to the participant's profile
Following	The outward network connections, i.e., other Instagram profiles that the participant subscribed to
Requested	The outward network connections which are not confirmed yet, i.e., other private Instagram profiles that the participant subscribed to, but which have not been confirmed by the other party yet
Follower-Following Ratio	The ratio of number of followers divided by number of following

#### 2.1.1. Data Extraction, Exclusion, and Subject Matching

The Instagram archive data was parsed and aggregated. We included the entire archive for analysis including pre and post diagnosis data as psychotic symptoms are known to emerge well in advance of receiving the diagnosis (41). Further, prior work has demonstrated that digital media traces on Facebook showed changes over a year before the initial diagnosis (24). We selected a minimum criteria of at least 5 posts over a timespan of at least 3 months—a timespan previously used as a time window for classification tasks (24). This resulted in the exclusion of 11 participants with SSD and 12 HVs. To ensure comparability of the SSD and HV groups, we created two equally sized samples with a comparable distribution of age, sex, and race attributes. Each SSD participant was matched to a HV participant based on these three covariates. We used the “Matching” R package (42), which provides functions for finding optimal covariate balancing in samples based on propensity scores, for selecting participants from the HV group to match the SSD participants. The characteristics of the final set of participants can be found in Table 2.

**Table 2:** Overview of participants.

	**Total** **(*n* = 68)**	**HV** **(*n* = 34)**	**SSD** **(*n* = 34)**
**Age**	23.6 (± 4.1)	23.5 (± 3.8)	23.6 (± 4.4)
**Sex**			
Female	28 (41%)	16 (47%)	12 (35%)
Male	40 (59%)	18 (53%)	22 (65%)
**Race**			
African American	22 (32%)	7 (21%)	15 (44%)
Native American	16 (24%)	10 (29%)	6 (18%)
Other/mixed	6 (9%)	3 (9%)	3 (9%)
White	24 (35%)	14 (41%)	10 (29%)
**Ethnicity**			
Hispanic	9 (13%)	5 (15%)	4 (12%)
Non-Hispanic	59 (87%)	29 (85%)	30 (88%)
**Diagnosis**			
Schizophrenia spectrum disorder	14 (21%)	0 (0%)	14 (41%)
Schizoaffective disorder	5 (7%)	0 (0%)	5 (15%)
Schizophreniform	9 (13%)	0 (0%)	9 (26%)
Unspecified schizophrenia spectrum disorder	6 (9%)	0 (0%)	6 (18%)
**Instagram usage**			
Instagram Usage (months)	48.8 (± 26.2)	56.4 (± 23.0)	41.2 (± 27.3)
Average Instagram Posts per Month	4.8 (± 10.6)	3.6 (± 6.4)	6.0 (± 13.5)

*Demographic and basic Instagram usage overview*.

#### 2.1.2. Image Processing and Analysis

Our analysis was aimed at investigating differences in color composition of the participants Instagram posts. Images were processed using the Python OpenCV library (43). Image statistics, such as width, height and aspect ratio, were extracted; see Table 3 for details. We compared the aspect ratio—defined as the ratio of width to height of a photo—of the images. Of note, the feature that allowed users to alter the aspect ratio of an uploaded image was introduced in the Android and iOS app in mid 2015 (44). To account for users who updated late to this app version, we did not include posts before January 2016 in the aspect ratio analysis.

**Table 3:** Overview of differences in image characteristics.

	**HV (*n* = 34)**			**SSD (*n* = 34)**						
**Feature**	**Mean**	**SD**		**Mean**	**SD**	***p***	***df*^†^**	**Cohen's** ***D***	**CI 95%**	**Hedges** ***g***
**Face detection**										
Number of Faces	2.38	1.01		1.46	0.79	**0.000**		–1.03	[–1.75, –0.31]	–1.02
**Image dimension and aspect ratio**										
Width (px)	1017.45	46.36		976.18	78.34	**0.025**		–0.65	[–1.34, 0.04]	–0.64
Height (px)	1038.46	68.47		1008.34	108.99	0.183	53.59	–0.34	[–1.01, 0.34]	–0.33
Aspect ratio	1.02	0.05		1.00	0.08	0.089		–0.24	[–0.91, 0.44]	–0.23
Non-square images	56.83	27.53		65.72	28.45	0.166		0.32	[–0.36, 1.00]	0.32
**Color features**										
Average R	127.41	12.51		124.82	18.74	0.505	57.53	–0.16	[–0.83, 0.51]	–0.16
Average G	115.85	12.28		111.62	16.63	0.237	60.74	–0.29	[–0.96, 0.39]	–0.29
Average B	110.57	12.03		105.68	15.77	0.060		–0.35	[–1.02, 0.33]	–0.34
Variance R	66.80	4.25		65.71	7.41	0.222		–0.18	[–0.85, 0.49]	–0.18
Variance G	63.97	5.03		63.25	6.70	0.619	61.21	–0.12	[–0.79, 0.55]	–0.12
Variance B	60.88	4.95		60.08	6.22	0.557	62.83	–0.14	[–0.82, 0.53]	–0.14
Skewness R	0.06	0.25		0.18	0.80	0.966		0.19	[–0.48, 0.86]	0.19
Skewness G	0.22	0.28		0.42	0.82	0.354		0.32	[–0.35, 1.00]	0.32
Skewness B	0.38	0.26		0.59	0.80	0.323		0.34	[–0.34, 1.02]	0.34
Average H	30.98	14.66		25.30	15.79	0.129	65.64	–0.37	[–1.05, 0.31]	–0.37
Average S	83.9	9.88		77.63	13.53	**0.033**	60.41	–0.53	[–1.21, 0.16]	–0.52
Average V	138.82	12.26		132.06	17.78	0.073	58.58	–0.44	[–1.12, 0.24]	–0.44
Variance S	50.10	5.09		47.34	8.01	0.096	55.89	–0.41	[–1.09, 0.27]	–0.41
Variance V	65.34	4.09		65.11	7.08	0.458		–0.04	[–0.71, 0.63]	–0.04
Skewness S	0.88	0.72		0.84	0.50	0.429		–0.06	[–0.73, 0.61]	–0.06
Skewness V	–0.08	0.27		0.10	0.77	0.241		0.31	[–0.37, 0.99]	0.31
Colorfulness	41.86	5.51		37.25	7.51	**0.005**	60.54	–0.70	[–1.39, 0.00]	–0.69

*Significant (p < 0.05) differences are highlighted in bold*.

† *Degrees of freedom (df) are reported where t-tests have been performed*.

For each image, a histogram analysis of two color spaces commonly used in image analysis were performed (45). These color spaces provide three values for each pixel in the range from 0 to 255 (apart from hue, which is in the range of 0 to 179 in OpenCV):

- The *red-green-blue (RGB)* color space is an additive model where each pixel's color is divided into red, green, and blue components. The higher the value for a color component, the higher it contributes to the resulting pixel color, e.g., a purple pixel will have a high red and blue component but a low green component. This model was chosen to identify the prevalence of these basic color components in the photos. It has been successfully implemented in prior research exploring color composition of photos posted on Instagram (46).
- The *hue-saturation-value (HSV)* color space is closely related to human perception of colors (47). Each pixel's color is represented by a hue, a saturation, and value component. The value component represents the illumination of the color: a low value means that the color is dark, while a high value represents a fully illuminated color. In prior work, the HSV model has been successfully used to explore color composition of Instagram posts (19, 48).

**Figure 1:**
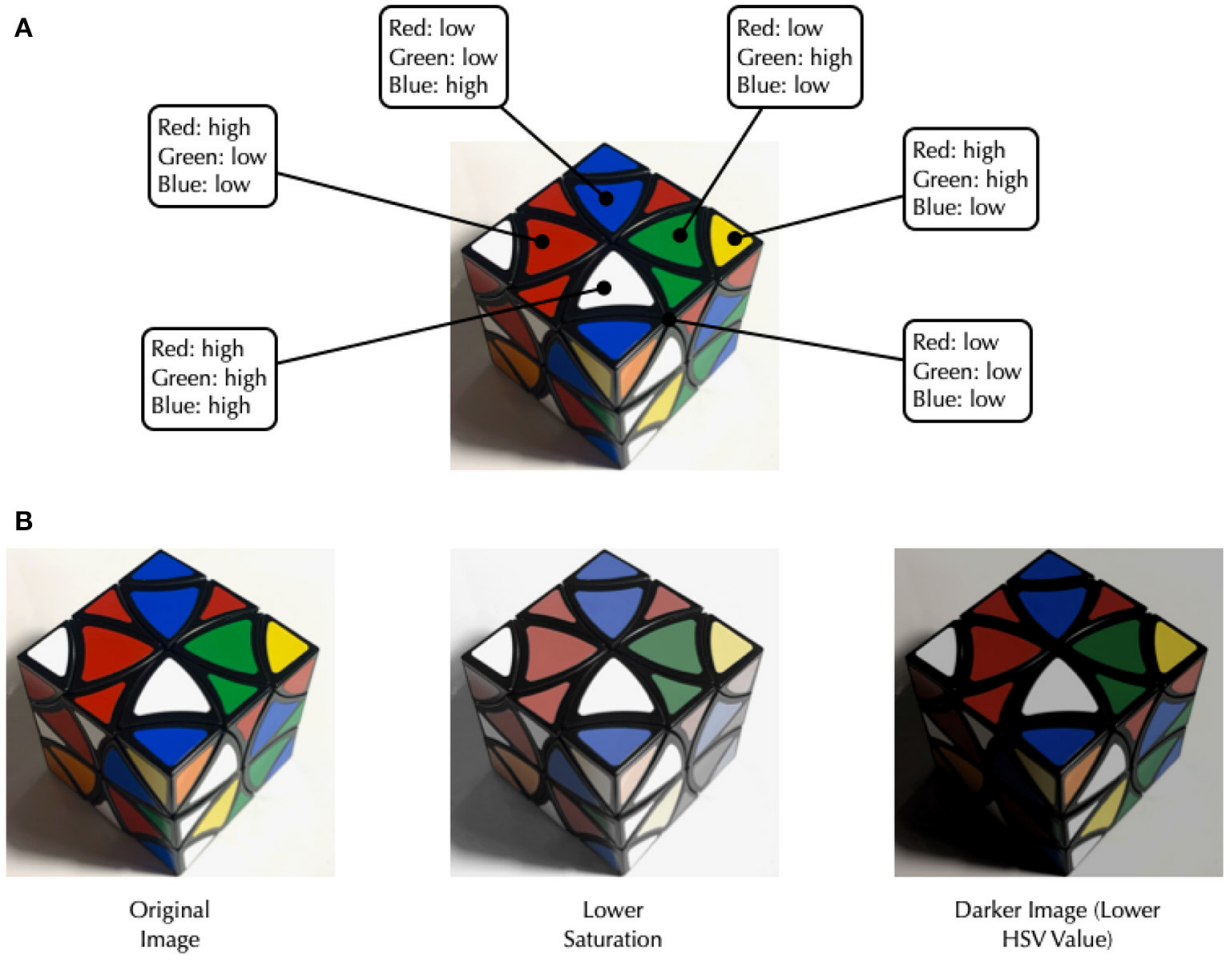
Illustration of color characteristics in images. **(A)** Illustration of the red-green-blue RGB color space. **(B)** Illustration of lower saturated and darker images.

The RGB and HSV color space are illustrated in Figure 1. For each image, we calculated the average, standard deviation and skewness of each color space component, apart from hue. Since hue is a radial value, meaning high values in hue have a short distance to low values, we used the mean of circular quantities.

To assess the colorfulness of each image, we implemented the metric proposed by Hasler et al. (49). They identified a model to fit human-rated colorfulness scores of images. Prior work has related this metric to agreeable personality traits on Instagram (46).

To detect and count the presence of human faces, a Haar-Cascade classifier from the OpenCV library (43) was used. The count and bounding box areas of each detected face were tracked and analyzed.

#### 2.1.3. Analysis of Network Connections

Most online social networks offer the ability to form connections with other users of the platform. While certain platforms such as Facebook use bidirectional connections called *friendships*, Instagram implements unidirectional edges between its users. This means that users who subscribe to another user's content, i.e., become *followers*, are not automatically followed back by said user. Each user, therefore, has outwards connections, called *following*, and inward connections, called *followers*. This can lead to imbalance dynamics where users with a high number of followers can push their content to a large number of people on the social network (commonly termed influencers). Following guidance from De Choudhury et al. (18), we analyzed the ratio of followers to following connections as well as the percentage of connections per type, i.e., followers, following, follow requests, etc., per participant.

#### 2.1.4. Temporal Aspects of Posting Behavior

We extracted timestamps for each post. These posts were grouped based on their time during the day. We created 4 categories: morning (6 a.m.–12 p.m.), afternoon (12 p.m.–6 p.m.), evening (6 p.m.–12 a.m.), and night (12 a.m.–6 a.m.); as proposed by De Choudhury et al. (18) and Feuston and Piper (50) to try to detect changes in circadian rhythm, which commonly occur in individuals with mood disorders or SSD (51). We further extracted the day of the week from posts.

#### 2.1.5. Statistical Analysis

All of the above described metrics were averaged for each participant. Descriptive and inferential statistics were used to explore and analyze each metric. Normal distribution was checked using Shaipiro-Wilk and visual inspection of QQ-Plots. Consequently, independent *t*-tests or non-parametric Mann-Whitney *U* tests (in the case of non-normality or the existence of extreme outliers [see Zimmerman (52)]) were performed to establish between-group differences. The *t* statistic with degrees of freedom (*df*) in case of independent *t*-test or the *U* statistic in case of Mann-Whitney *U* tests are reported below.

We matched participants for their sex, age, and race. Due to a slight imbalance in sex, we performed *t*-tests or non-parametric Mann-Whitney *U* tests (in the case of non-normality) over all features to control for sex-related differences. In case of significant differences, we performed a secondary analysis in form of a multiple linear regression to investigate the impact of group, sex, age, and race on the feature.

To explore temporal differences across the times of the day (night, morning, afternoon, evening) and days of the week, we performed mixed factorial ANOVA. Homogeneity of variance was tested using Levene's test. Due to violation of the pre-requisites, robust mixed factorial ANOVA (53) was performed using the R package WRS2 (54). All statistical tests were performed using R (Version 3.6.3).

## 3. Results

We analyzed a total of 11,947 images spanning 3–91 months across 68 participants with SSD (*n* = 34) and HV (*n* = 34). An overview of the participant characteristics is depicted in Table 2. The average usage duration (characterized as the timespan between the first and latest post) was 56.4 months for HV and 41.2 months for SSD. On average, the HV group posted 3.6 (*SD* = 6.4) posts compared to 6.0 (*SD* = 13.5) posts per month in the SSD group.

### 3.1. Image Characteristics

We analyzed within-participant averaged image characteristics including colorimetry, colorfulness, face count, and aspect ratio. A summary of the image results, including group mean, standard deviation, *p* value, and Cohen's *D* is shown in Table 3.

**Figure 2:**
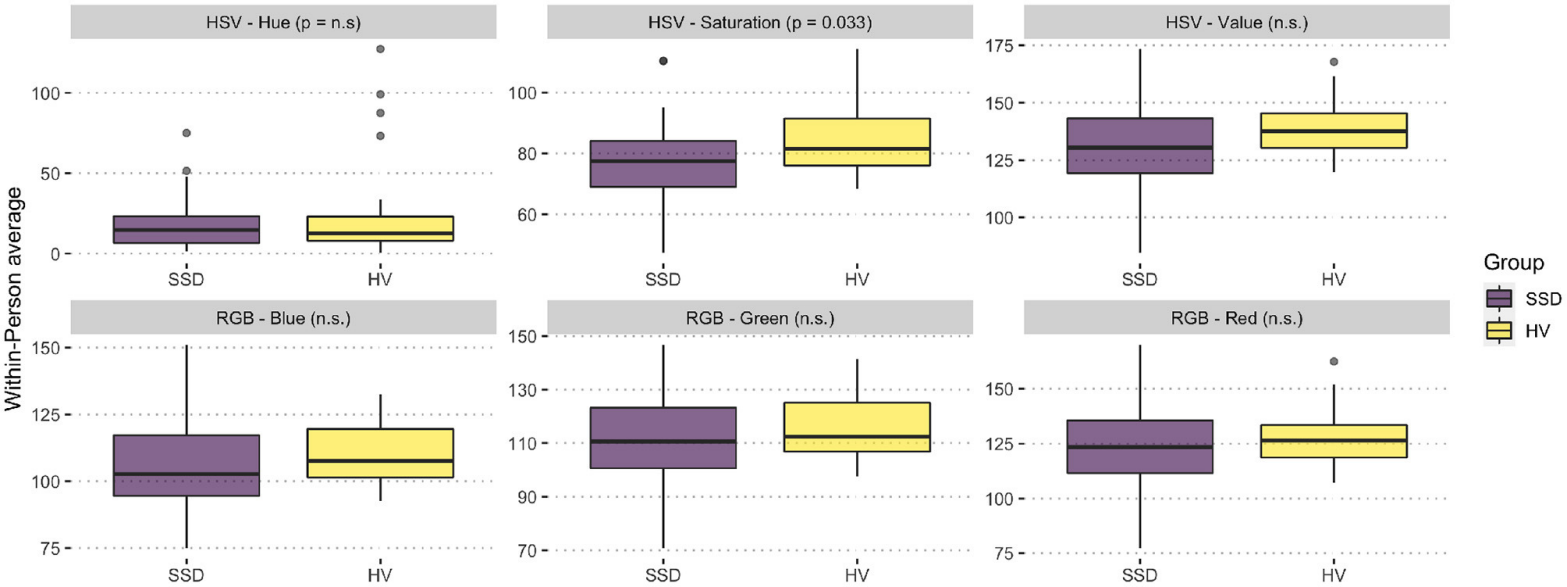
Overview of HSV and RGB color distribution in image posts. Pairwise Comparison of the color components of the HSV and RGB color spaces. Results show significantly higher average saturation in HV compared to SSD, but no significant (n.s.) differences in all other color components.

The analysis of average colors revealed a significantly lower average saturation in pictures posted by participants with SSD compared to HV [*t*_(60.41)_ = –2.182, *p* = 0.033; SSD: Mean = 77.63, *SD* = 13.53; HV: Mean = 83.9, *SD* = 9.88]. This indicates a higher prevalence of vibrant colors in the posts of HVs compared to more muted colors for the SSD group (see Figure 1). Although not significant, the HSV value component showed a tendency to be lower amongst individuals with SSD [*t*_(58.58)_ = –1.824, *p* = 0.07] indicating a trend toward darker images. We did not identify any significant difference in the hue component. Further, we did not identify significant differences in the average RGB color components. The analysis of the average standard deviation, i.e., the variance, and skewness of color in an image, revealed no significant differences between both groups. An overview of the color components per group can be found in Figure 2. The analysis of colorfulness (49) revealed that individuals with SSD posted less colorful images compared to HV [*t*_(60.54)_ = –2.8869, *p* = 0.0054, SSD: Mean = 37.25, *SD* = 7.51; HV: Mean = 41.86, *SD* = 5.51].

We counted the average number of human faces detected in the posted Instagram photos. Comparing both groups, we found individuals with SSD shared images which on average contained less faces than the HV group (*U* = 264.5, *n*_1_ = *n*_2_ = 34, *p* < 0.001; HV: Mean = 2.38, *SD* = 1.01; SSD: Mean = 1.46, *SD* = 0.79). There was no statistical difference in aspect ratio (the ratio of width compared to height of an image) between the SSD group (Mean = 1.00, *SD* = 0.09) and HV group (Mean = 1.02, *SD* = 0.05); *U* = 426, *n*_1_ = *n*_2_ = 34, *p* = 0.091. Since the default aspect ratio of Instagram posts is 1:1 (squared), for each participant, we also compared the percentage of posts where the aspect ratio was diverting from the default—indicating deliberate changing of the aspect ratio by the user. There was no difference in the percentage of posts where the aspect ratio diverted from the default (*U* = 605.5, *n*_1_ = *n*_2_ = 34, *p* = 0.438; SSD: mean = 44.8%, *SD* = 28.8; HV: mean = 34.3%, *SD* = 28.4).

Due to a slight imbalance in the male-female ratio between the groups, we tested each image characteristic for sex differences. We did not find any impact of sex on the color composition or aspect ratio. However, we identified a difference in face count based on sex (*U* = 742; *n*_*f*_ = 28, *n*_*m*_ = 40, *p* = 0.024) suggesting that women had on average more faces in their Instagram posts (female: Mean = 2.15, *SD* = 0.84; male: Mean = 1.76, *SD* = 1.1). To further explore this finding, we performed a secondary analysis in the form of a multiple linear regression. Results of the multiple linear regression to predict face count indicated that there was a collective significant effect between the group (HV or SSD), sex, age, and race [*F*_(6,61)_ = 5.588, *p* < 0.0001, *R*^2^ = 0.35]. However, the individual predictors were examined further and indicated that group (*t* = 3.615, *p* = 0.0006) and age (*t* = –2.502, *p* = 0.005) were significant predictors in the model and that sex was not significant (*p* = 0.360). Given that age was matched between both groups, we concluded that the differences in face count are indeed related to differences between the SSD and HV groups and not sex.

### 3.2. Social Connections to Other Users

We analyzed the in- and outward connections of each participant with other Instagram users. When we normalized the number of connections by the usage duration, we did not find a significant difference between the SSD and the HV groups. We then, however, calculated the ratio by dividing the number of followers by the number of following, and did identify significant differences (*U* = 760, *n*_1_ = *n*_2_ = 34, *p* = 0.025). Specifically, the SSD group had a lower proportion of followers per following (SSD: Mean = 0.88 followers per following, *SD* = 0.51; HV: Mean = 1.33 followers per following, *SD* = 1.3; Cohen's *D* = –0.46, 95% CI [–1.14,0.22]). This trend seems to be driven by the number of followers in particular (SSD: Mean = 416.0, *SD* = 352.87; HV: Mean = 906.94, *SD* = 1565.53; Cohen's *D* = –0.43, 95% CI [−1.11,0.25]) which was significantly different between the groups (*U* = 780, *n*_1_ = *n*_2_ = 34, *p* = 0.013). To assess if the number of followers is dependent on the duration of use of the platform, we analyzed the correlation between months of Instagram usage and followers and found a weak relationship using Spearman correlation (ρ_*S*_ = 0.239, *p* = 0.049). Comparatively, there was no correlation between the follower-following ratio and duration of use (*p* = 0.601) rendering it a less biased metric. A detailed overview of these results is depicted in Table 4.

**Table 4:** Statistics of network connections.

	**HV (*n* = 34)**			**SSD (*n* = 34)**					
**Feature**	**Mean**	**SD**		**Mean**	**SD**	***p***	**Cohen's** ***D***	**CI 95%**	**Hedges** ***g***
**Absolute number of connections**									
Follower	906.94	1565.53		416.00	352.87	**0.013**	–0.43	[–1.11, 0.25]	–0.43
Following	640.82	464.31		599.91	697.23	0.168	–0.07	[–0.74, 0.60]	–0.07
Request	9.97	16.49		12.09	12.88	0.263	0.14	[–0.53, 0.82]	0.14
Hashtag	0.76	2.32		4.35	11.32	0.359	0.44	[–0.24, 1.12]	0.43
Blocked	5.82	9.71		8.03	16.86	0.353	0.16	[–0.51, 0.83]	0.16
Close	10.91	33.03		2.50	8.25	0.313	–0.35	[–1.02, 0.33]	–0.35
Restricted	0.03	0.17		0.03	0.17	1.000	0.00	[–0.67, 0.67]	0.00
**Connections normalized per usage duration in months**									
Blocked (normalized)	0.17	0.40		0.44	1.58	0.499	0.24	[–0.44, 0.91]	0.23
Close (normalized)	0.20	0.53		0.08	0.31	0.362	–0.28	[–0.96, 0.39]	–0.28
Follower (normalized)	23.70	44.81		20.82	33.77	0.285	–0.07	[–0.74, 0.60]	–0.07
Following (normalized)	16.14	22.82		21.74	26.45	0.650	0.23	[–0.45, 0.90]	0.22
**Relative number of connections**									
Followers/following	1.33	1.30		0.88	0.51	**0.025**	–0.46	[–1.14, 0.22]	–0.46
Request/following	0.02	0.02		0.03	0.05	0.105	0.45	[–0.24, 1.13]	0.44

*Significant (p < 0.05) differences are highlighted in bold. Followers represent other users followed by the participant; Following represent other Instagram users who follow the participant; Requests represent a request to follow a private account has been initiated by the participant; Hashtags indicates that the participant subscribed to updates from a hashtag; Blocked means that the participant blocked other Instagram accounts from seeing his/her updates; Close means that the participant marked another Instagram account as a close friend (selectively, some content can just be shared with close friends); Restricted means that a participant chose to restrict commenting functionality for another Instagram account*.

### 3.3. Temporal Behavioral Characteristics

**Figure 3:**
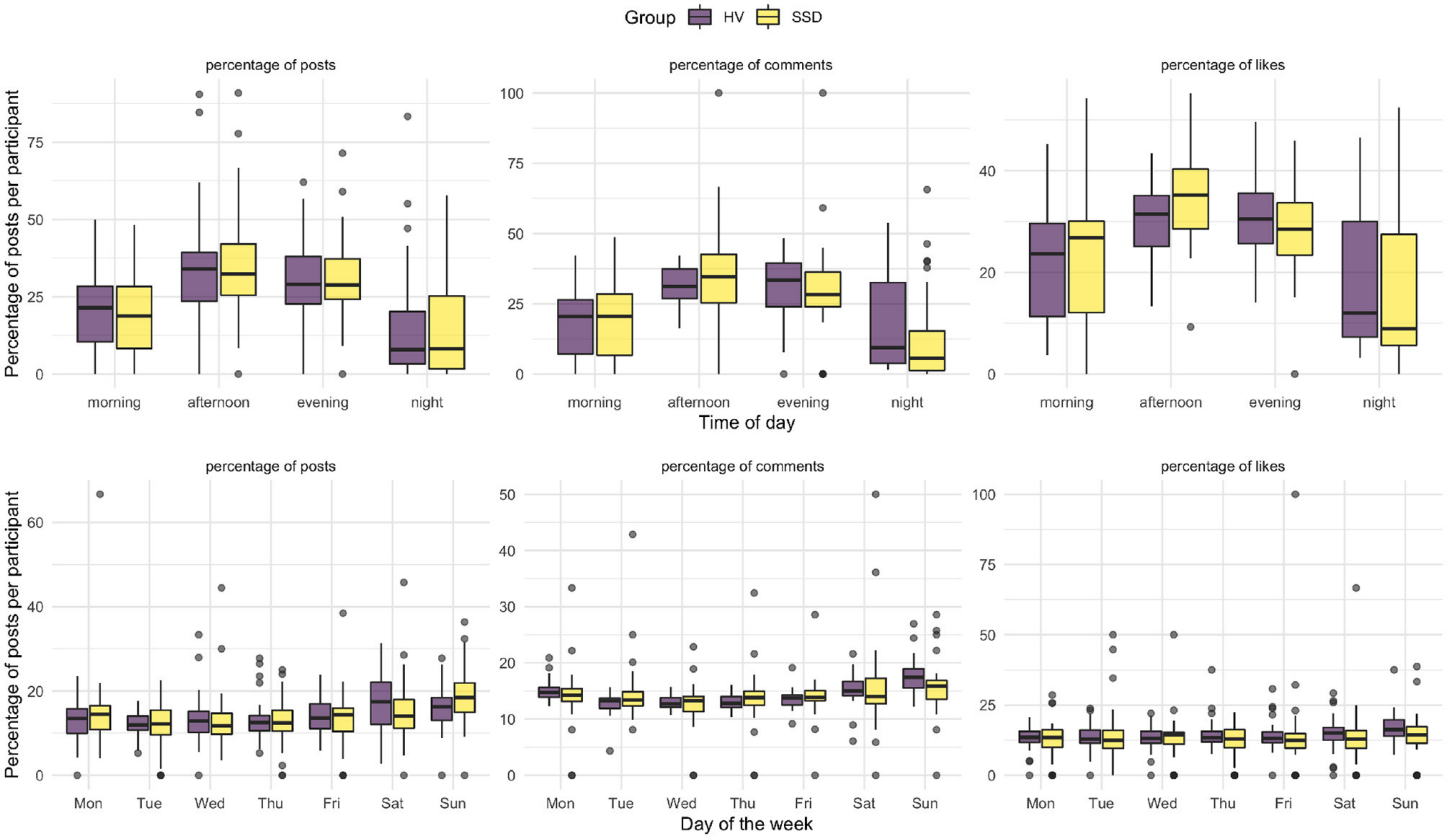
Temporal aspects of posting behavior. The graphics illustrate an overview of the percentage of actions (i.e., posting content, liking other user's content, commenting on other user's content) per participant that have been performed in each of these temporal categories. Top plot: Posting behavior throughout the day: The data was binned into morning (6 a.m.–12 p.m.), afternoon (12 p.m.–6 p.m.), evening (6 p.m.–12 a.m.) and night (12 a.m.–6 a.m.) in accordance with Feuston and Piper (50). Bottom plot: The plot shows the percentage of posts during the days of the week.

To explore temporal differences in posting behavior during the time of day and days of the week, we performed a robust factorial mixed ANOVA using the WRS2 R package (54) due to the non-normality and violation of homogeneity of covariance in the data. We compared the main effects of the within-subject variable *day of the week*, e.g., Monday, Tuesday, etc., and the between-subject variable of *group* on the posting frequency. We also compared interaction effects between *day of the week* and *group*. We identified a main effect of the *day of the week* on Instagram behavior [*Q*_(6,30.12)_ = 12.24, *p* < 0.001]. Specifically, there was an increase in posting behavior on Saturdays and Sundays for both groups. We did not identify a main effect for *group* [*Q*_(1,30.56)_ = 2.77, *p* = 0.107] nor an interaction between *day of the week* and *group* [*Q*_(6,30.12)_ = 1.81, *p* = 0.132]. This suggests that diagnosis does not affect temporal posting behavior during the course of the week. Similarly, we compared main effects of the within-subject variable of *times of the day*^1^ and the between-subject variable of *group* on the posting frequency. We also compared interaction effects between *time of the day* and *group*. We found main effects for the posting behavior during *times of the day* [*Q*_(3,43.92)_ = 13.77, *p* < 0.001]. However, we did not find main effects for the *group* [*Q*_(1,86.54)_ = 0.01, *p* = 0.923] nor interaction effects between *time of the day* and *group* [*Q*_(3,44.0)_ = 0.37, *p* = 0.775]. This indicates that diagnosis does not affect temporal posting behavior throughout the day. In summary, no differences in posting behavior between the SSD and HV groups were found. An overview of posting behavior throughout the day and days of the week can be seen in Figure 3.

## 4. Discussion

In this paper, we explored differences in usage patterns between participants with schizophrenia spectrum disorder (SSD) and non-clinical healthy volunteers (HV) on Instagram. We focused on the characteristics of the images posted, online behavior, and social connections to other platform users. We identified several features in images and social networks that successfully distinguished between the SSD and HV groups. Our work contributes by investigating Instagram data donated directly by consented participants receiving psychiatric care with established psychiatric diagnoses.

### 4.1. Characteristics of Image Posts on Instagram

We found that, on average, participants with SSD uploaded images that were less colorful, less saturated and, although not significant, showed a tendency to be darker^2^. Prior research on color preference has shown that the presence of a psychiatric disorder can influence what colors individuals find appealing in images (55, 56). Still, more research is needed to determine how and if color preferences impact the selection of images uploaded to social media. While some work has demonstrated no differences in color preference compared to healthy participants (57), others have shown a preference for the color “red” and “black” (58). When considering images uploaded to social media by individuals with mental health concerns, there has been limited prior work. Birnbaum et al. (24) found lower contrast in the photos posted to Facebook by participants with SSD and mood disorder (MD) compared to HV. They also identified a tendency for the MD group to post bluer and more yellow photos. Yazdavar et al. (59) reported that profile pictures of Twitter users with self-disclosed depression had a more gray, less saturated, and overall less appealing color characteristics compared to HVs. Along similar lines, Reece et al. (19) showed that individuals with depression posted images on Instagram that were lower in color saturation and darker and had a higher hue (toward blue). In contrast, Manikonda and De Choudhury (37) observed that Instagram images with hashtags related to mental illness had high saturation, however, it is unknown if these images were posted by individuals with psychiatric disorders themselves.

Our results contribute to the existing body of work by demonstrating that individuals with SSD post images with similar color composition as individuals with depression on Instagram (19). One possibility is that altered color composition may represent a marker of mental illness that is being expressed in the selection of images uploaded to Instagram. Importantly, prior findings are not consistent across platforms. Further research will be required to explore how image characteristics and color composition correlate with a presence of psychiatric illness and to determine the extent to which differences are unique to Instagram or consistent across platforms. Importantly, the consideration of aggregated and summary features does not give insights into the underlying reasons for these differences, such as filter use, differences in image content, and even differences in the intent and motivations behind the use of these platforms for image sharing. Our future work aims to explore color composition in different regions of the image, e.g., comparing background and foreground colors or colors of salient regions (60) to identify deeper signals of image differences.

When considering the width, height, and aspect ratio (the ratio of width to height), we did not find any differences between the two groups. This is contrary to findings by Birnbaum et al. (24) who analyzed images posted to Facebook and found that participants with SSD and MD posted images that were smaller in width and height than HV posts. Of note, different social media platforms offer unique image manipulation options and affordances, which could be one explanation for differing results. For instance, Instagram is focused on visual aesthetics and provides an easily accessible set of filters which are not available on Facebook (31). Further research will be necessary to understand how participants with psychiatric disorders use the various image manipulation options to express themselves and how these patterns correlate with illness and health.

In line with prior work comparing individuals with depression to healthy volunteers on Instagram and Twitter (19, 59), we found that on average SSD participants uploaded images with a lower average face count. Interestingly, both individuals with SSD and those with depression may experience social isolation and/or withdrawal, especially during symptomatic exacerbations. Reduced face count in uploaded images could therefore represent a digital expression of reduced social interactions and contacts. Although not specifically explored here, an important question for future research would be to assess differences in face count throughout illness progression and exacerbation as symptoms, and social interactions, fluctuate over time. Further, prior work has demonstrated the significance of posting self-portraits (selfies, for example) as a non-verbal tool of communicating intention from sender to receiver (61). Accordingly, future work should consider exploring potential differences in the use of selfies, as well as other metrics such as the face to background distribution, facial angle, and facial expressions in individuals diagnosed with SSD.

### 4.2. Social Connections to other Instagram Users

We found that participants with SSD had a different makeup of network connections compared to HV. While, the average number of connections acquired per month, e.g., followers divided by the duration of use, was not significantly different, considering ratio-based metrics showed a significant difference. Specifically, the ratio of followers to users they followed was skewed toward a lower proportion of followers. This is consistent with the observations of De Choudhury et al. (18) who examined the social network structures of depressed individuals on Twitter and found their ego-networks to be smaller with low reciprocity from directed social interactions. One explanation could be that Instagram profiles of individuals with SSD attract less followers, possibly due differences in image/color characteristics or shorter usage duration, making their profiles less attractive to other users. Additionally, we showed that the participants with SSD had a higher proportion of follow requests that were sent to other users, and were still waiting to be accepted, suggesting that other Instagram users are less likely to accept follow requests from individuals with SSD. Of note, work by Birnbaum et al. (20) found that individuals with SSD showed increased befriending behavior on Facebook in the month preceding a relapse hospitalization and they argued that this could represent disorganized social behavior, often seen in individuals with worsening psychosis (62, 63). Though further research is required, extracting social media-based interaction data from individuals with SSD may one day offer valuable clinical insight related to social interactions.

### 4.3. Temporal Aspects of Behavior

In contrast to prior work demonstrating disturbances in circadian rhythm in individuals with mental illness (51) reflected by an increased night-time online activity (18, 24, 64), we did not identify any temporal behavioral changes on Instagram in SSD. This may be partially explained by the fact that circadian rhythm disruptions in individuals with SSD are typically most apparent during symptomatic exacerbations. Of note, we extracted and analyzed the entire timeline, which may have included images uploaded prior to developing clinically significant symptoms of SSD as well as during periods of relative health. Future work should consider monitoring participants prospectively with symptom rating scales and exploring changes that occur on Instagram when symptoms are most prominent and circadian rhythm disruptions most severe.

### 4.4. Limitations

Several important limitations are worth noting. Firstly, the sample size was relatively small as compared to prior working exploring Instagram activity in mental health and may therefore limit generalizability. However, our work contributes as it represents the first to leverage data uploaded and donated by real patients receiving psychiatric care for schizophrenia. Additionally, there may be characteristics in Instagram usage patterns of individuals with SSD that were not recruited or declined participation that were not captured in our dataset. Future studies using larger samples are necessary to confirm our findings and support generalizability. Second, our eligibility criteria limited the age range to those between 15 and 35 years, and it is unclear at this time if younger adolescents or older adults engage differently with Instagram than our participants. Third, archives included retrospective data including images uploaded prior to (and after) receiving a psychiatric diagnosis. It is unclear how long the participants in our dataset were experiencing psychiatric symptoms and to what extent active psychiatric symptoms, hospitalization, receiving a psychiatric diagnosis, or ongoing treatment impact Instagram activity. Finally, some of the findings may relate to use of technology that is not represented in Instagram archives. For example, prior work related image filter usage to psychiatric diagnosis (19), something that was not feasible with the data presented in the Instagram archives. Further, factors such as camera specifics in the mobile phone can impact image resolution, color, and quality (65) and were not available for analysis, and more sophisticated image analysis approaches, such as those using recent advances in deep learning and convolutional neural networks, were not explored due to a limited ability to meaningfully interpret the results. Collecting additional data about phone models and testing more sophisticated image feature extraction might inform future work.

### 4.5. Conclusion

Images play an important role in online self expression. Due to the wide availability and usage of online social networking sites, image sharing platforms like Instagram propose an intriguing medium for prediction, identification, and monitoring of serious mental illnesses, including schizophrenia spectrum disorder. Much like physicians routinely order X-rays and blood tests to inform medical care, Instagram data could one day be incorporated into psychiatric assessments as an objective source of collateral data used to inform diagnostic procedures and clinical decision making. Utilizing Instagram data as collateral information would represent a major advancement in efforts to capitalize on objective digital data to improve outcomes. This would be a significant step forward for psychiatry, which has historically been limited by its reliance on self-report.

## Data Availability Statement

The datasets presented in this article are not readily available because of participant privacy and security concerns, including HIPAA regulations. Requests to access the datasets should be directed to Mbirnbaum@northwell.edu.

## Ethics Statement

The studies involving human participants were reviewed and approved by the Northwell Health Institutional Review Board. Written informed consent to participate in this study was provided by the participants' legal guardian/next of kin.

## Author Contributions

MB, MDC, and JK conceptualized and executed the project and interpreted the results. MB, SY-C, and WM contributed to participant recruitment and data collection. KH conducted the data analysis along with support from IL and MS. KH and MB wrote the initial draft of the manuscript and all other authors contributed to the manuscript preparation and editing.

## Conflict of Interest

The authors declare that the research was conducted in the absence of any commercial or financial relationships that could be construed as a potential conflict of interest.

## Publisher's Note

All claims expressed in this article are solely those of the authors and do not necessarily represent those of their affiliated organizations, or those of the publisher, the editors and the reviewers. Any product that may be evaluated in this article, or claim that may be made by its manufacturer, is not guaranteed or endorsed by the publisher.

## Footnotes

^1^For the posting behavior throughout the day, we chose 4 time windows in accordance with De Choudhury et al., (18), Feuston and Piper (50), and Birnbaum et al. (24). These 4 time windows are: morning (6 a.m.–12 p.m.), afternoon (12 p.m.–6 p.m.), evening (6 p.m.–12 a.m.), and night (12 a.m.–6 a.m).

^2^For an example of how darker and less saturated images appear, please refer to Figure 1.
